# Ultrafast Reactive Laser Sintering of Highly Conductive Garnet-Type LLZTO Solid Electrolytes

**DOI:** 10.1021/acsami.6c05262

**Published:** 2026-07-06

**Authors:** Erika P. Ramos, Yudan Chen, Aaron Santomauro, Yan-Yan Hu, Samuel Sanghyun Lee, Jianhua Tong, Jianchao Ye

**Affiliations:** † Materials Science Division, 4578 Lawrence Livermore National Laboratory, Livermore, California 94550, United States; ‡ Department of Chemistry & Biochemistry, 7823 Florida State University, Tallahassee, Florida 32306, United States; § Department of Materials Science and Engineering, 2545 Clemson University, Clemson, South Carolina 29634, United States; ∥ Mechanical Engineering, 6429 Stanford University, Stanford, California 94305, United States

**Keywords:** reactive laser sintering, garnet solid electrolytes, solid-state batteries, ultrafast densification, CO_2_ laser

## Abstract

Rapid and scalable fabrication of garnet-type solid electrolytes remains a major challenge for the practical deployment of lithium metal batteries. Here, we report reactive laser sintering (RLS) as an ultrafast and potentially scalable strategy for fabricating garnet-type Li_6_._4_La_3_Zr_1_._4_Ta_0_._6_O_12_ (LLZTO) solid electrolytes. RLS of LLZTO enables simultaneous reaction and densification, achieving ∼95% relative density while minimizing lithium loss and suppressing secondary phase formation. Compared to conventional furnace sintering, RLS promotes enhanced grain growth and improved densification, leading to improved ionic conductivity (0.36 ± 0.08 mS cm^–1^) while maintaining comparable activation energies for Li^+^ transport. Structural characterization by X-ray diffraction (XRD), Raman spectroscopy, and solid-state ^6^Li/^7^Li NMR confirms the formation of cubic garnet LLZTO with homogeneous microscale elemental distribution. In addition, nanoindentation measurements demonstrate that RLS preserves the mechanical properties of the garnet framework despite ultrafast localized thermal processing. By integrating simultaneous reaction and densification with tunable microstructural control, reactive laser sintering provides a promising manufacturing pathway for high-performance garnet solid electrolytes toward next-generation solid-state batteries.

## Graphical abstract

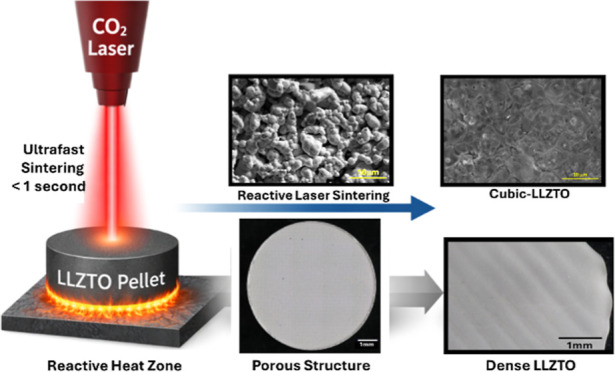

## 1 Introduction

Solid-state batteries (SSBs) are widely regarded as a promising next-generation energy storage technology because they offer improved safety and higher energy density than conventional lithium-ion batteries (LIBs).^1,2^ These advantages arise from the use of nonflammable solid electrolytes and compatibility with lithium metal anodes, which possess an exceptionally high theoretical capacity (∼3861 mAh g^–1^) and low redox potential (∼−3.04 V vs SHE).^3,4^ Together, these features enable substantially higher gravimetric and volumetric energy densities than conventional graphite-based LIBs, with projected values exceeding ∼500 Wh kg^–1^ and ∼1000 Wh L^–1^ in practical cell configurations.^5,6^ In addition, replacing flammable liquid electrolytes with solid-state electrolytes significantly improves operational safety by mitigating thermal runaway risks, which is particularly important for large-format and high-energy applications.^6,7^

Realizing these performance gains requires solid-state electrolytes that not only exhibit high ionic conductivity and electrochemical stability, but can also be fabricated by scalable, cost-effective manufacturing routes suitable for large-area production.^8,9^ Compared with sulfide-based electrolytes (e.g., Li_2_S–P_2_S_5_)^10–12^ and argyrodites (Li_6_PS_5_X)^13–15^ with high ionic conductivity but narrow electrochemical stability window, oxide-based Li_7_La_3_Zr_2_O_12_ (LLZO)^16^ and its doped derivatives (Nb, Ta,^17^ Ga, and Al)^18–20^ remain attractive because of their superior chemical robustness,^21^ broader electrochemical stability window with enhanced compatibility with both lithium metal and high-voltage cathodes.^22,23^ These characteristics position LLZO among the most promising solid electrolytes for practical solid-state battery applications.

Despite these advantages, scalable fabrication of dense LLZO remains a major challenge.^24^ Conventional high-temperature sintering (>1000 °C) typically requires prolonged dwell times, which promote lithium volatilization, nonuniform densification^25^ grain-boundary resistance, and secondary phase formation such as La_2_Zr_2_O_7_.^26,27^ Recent reviews have identified densification control, lithium retention, and interface stability as key bottlenecks for practical oxide solid electrolytes.^26,28,29^ Recent studies have shown that lithiophilic and electron-blocking interlayers can improve lithium wettability and suppress interfacial side reactions under stack pressure conditions,^30,31^ while scalable membrane fabrication approaches such as tape casting have enabled the production of dense garnet membranes for large-area solid-state battery architectures.^32^ These studies collectively demonstrate that both microstructural control and interfacial engineering are essential for practical implementation of LLZO-based electrolytes.

Considerable effort has therefore been devoted to developing alternative densification strategies capable of reducing thermal budgets while preserving lithium stoichiometry and achieving high relative density.^33–35^ Approaches including reactive sintering from pyrochlore precursors,^36,37^ molten-salt synthesis,^38,39^ oxygen-assisted sintering,^40,41^ hot pressing,^42^ cold sintering,^40,41^ and tape-casting-based rapid sintering routes^43^ have all demonstrated improved densification and microstructural control. More recently, ultrafast densification strategies such as spark plasma sintering (SPS),^44–46^ ultrafast high-temperature sintering (UHS),^47–49^ flash sintering,^25,50,51^ and Joule-heating-assisted processing^25,52^ have demonstrated dramatic reductions in processing time and thermal exposure for garnet electrolytes.^44,45,53,54^ SPS^51^ and hot-pressing methods can achieve high density and reduced grain-boundary resistance; however, these approaches typically require external pressure and conductive graphite tooling, limiting scalability and geometric flexibility.^46,54^ Flash sintering and Joule-heating-assisted methods enable densification within seconds but often rely on electric-field activation, conductive architectures, or presynthesized LLZO powders. Consequently, many rapid densification strategies still decouple garnet phase formation from densification,^23,55^ requiring separately synthesized LLZO powders prior to consolidation and maintaining energy-intensive precursor synthesis routes.^29,56,57^

In contrast, laser-based processing offers a pressureless and spatially selective route capable of generating highly localized heating with extremely high heating and cooling rates. Such localized thermal profiles can promote rapid densification while minimizing prolonged thermal exposure and lithium volatilization. In addition, the extreme thermal gradients and transient localized melting generated during laser exposure may enable rapid mass transport and melt-assisted densification pathways that are fundamentally distinct from equilibrium furnace-based processing. Such characteristics are particularly attractive for scalable and digitally controlled manufacturing of ceramic solid electrolytes. Building on this concept, we previously demonstrated ultrafast CO_2_ laser sintering of presynthesized LLZTO films within 1 s, achieving ∼95.7% relative density and ionic conductivity of 0.26 mS cm^–1^.^58^ However, most reported laser-based approaches still rely on presynthesized LLZO powders and therefore do not fully integrate phase formation and densification into a single processing step.

Here, we address these limitations through reactive laser sintering (RLS), a pressureless ultrafast processing strategy in which LLZTO precursors undergo simultaneous garnet phase formation and densification under highly localized laser heating in a controlled atmosphere. The resulting RLS LLZTO exhibits ∼95% relative density and enhanced ionic conductivity (0.36 mS cm^–1^), while minimizing prolonged lithium volatilization. Structural and chemical characterization using X-ray diffraction (XRD), Raman spectroscopy, solid-state ^6^Li/^7^Li NMR, and scanning electron microscopy (SEM) confirms cubic garnet formation, dense microstructures, and overall microscale compositional homogeneity. Direct comparison with conventionally furnace-sintered counterparts demonstrates that reactive laser sintering provides a rapid, scalable, and compositionally integrated pathway for manufacturing highly conductive garnet solid electrolytes.

## 2 Results and Discussion

A clear contrast exists between the traditional multistep fabrication of LLZTO ceramic electrolytes and the streamlined reactive laser sintering approach. Conventional processing typically involves repeated cycles of pelletization, calcination, and grinding or ball milling (Figure 1a), often performed multiple times to achieve the high density and ionic conductivity required for practical applications. These routes are time- and energy-intensive and can induce lithium loss, secondary phase formation, and significant challenges in scaling to large-area or high-throughput manufacturing. By contrast, reactive laser sintering, as shown in Figure 1b, offers a compelling alternative to conventional furnace sintering for LLZTO electrolytes. This method enables ultrafast sintering with subsecond dwell times, promotes high densification, mitigates lithium volatilization, and integrates phase formation and densification into a single processing step. To benchmark ultrafast reactive laser sintering (RLS) against conventional furnace sintering, ball-milled (BM) LLZTO precursors were pelletized and processed at 1100 °C for 6 h in a box furnace (Experimental Section).

**Figure 1:**
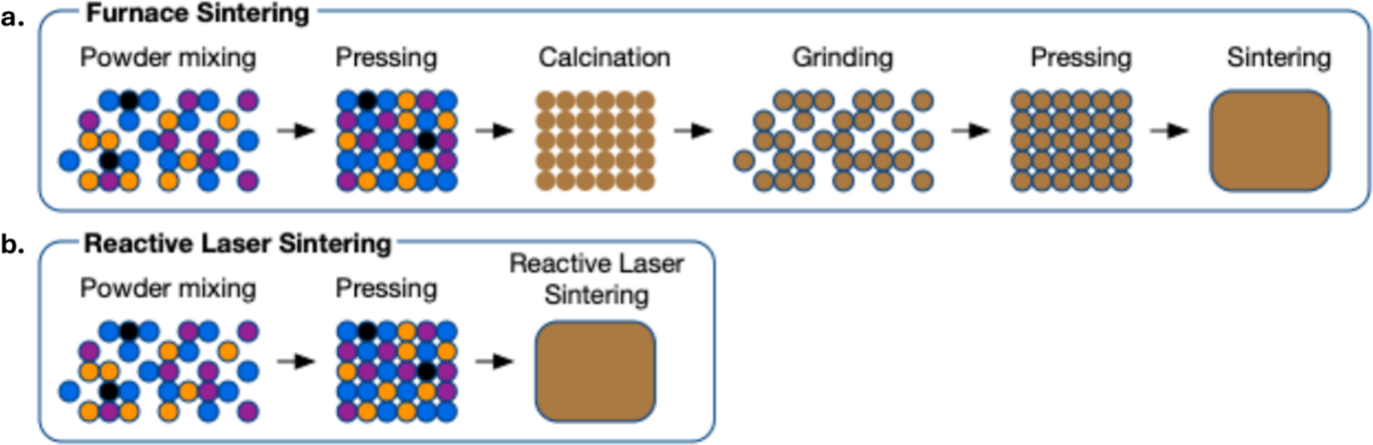
Schematic comparison of LLZTO ceramic electrolyte fabrication: (a) conventional multistep sintering involving repeated pelletization, calcination, and grinding; and (b) reactive laser sintering, which streamlines the process with fewer synthesis steps.

Figure 2 compares the microscopic appearance and surface profiles of pristine, furnace-sintered, and RLS-sintered LLZTO pellets. The pristine pellet (Figure 2a) is smooth and mechanically intact, with a diameter of ∼6.3 mm and a flat surface confirmed by optical profilometry. After furnace sintering (Figure 2b), the pellet diameter decreases to ∼5.92 mm and exhibits noticeable surface deformation, as revealed by profilometry. This behavior likely arises from nonuniform shrinkage during prolonged thermal treatment at 1100 °C for 6 h, leading to heterogeneous densification across the pellet.

**Figure 2:**
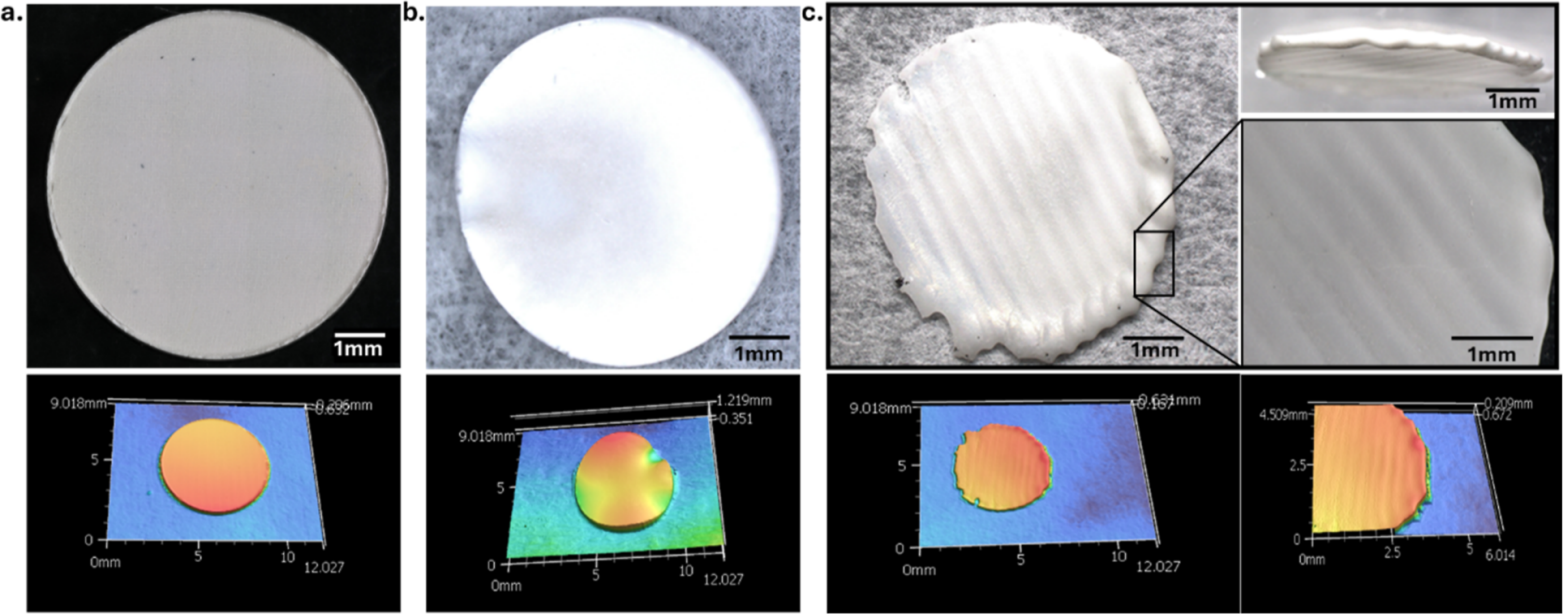
Macroscopic images and surface profiles of (a) pristine; (b) furnace-sintered, and (c) reactive laser sintered LLZTO pellets.

Unlike furnace sintering, which involves long-duration bulk heating with high cumulative thermal energy input, RLS operates under highly localized and transient energy delivery conditions characterized by high areal energy density. RLS LLZTO pellet (Figure 2c) was processed using an areal energy density (AED = *P*/*hu*) of 1.67 J mm^–2^ under a 2 W CO_2_ laser power (*P*), with a 1.5 mm (e^–2^) Gaussian beam diameter, scan speed (*u*) of 4 mm s^–1^, and hatch spacing (*h*) of 0.3 mm. These parameters were selected based on prior optimization in LLZTO CO_2_ laser sintering studies, where comparable energy densities enabled rapid densification under ultrafast thermal conditions.^58^ The RLS pellet shows a reduction in diameter from ∼6.0 mm to ∼5.5 mm, primarily attributed to localized edge rounding and minor material redistribution at the pellet perimeter during laser scanning. Despite this peripheral effect, the sample undergoes rapid densification within subsecond time scales and develops a glossy, laser-textured surface.

Surface profilometry reveals a periodic, wave-like morphology with an average amplitude of ∼0.2 mm, consistent with previously reported laser-processed LLZTO structures.^58^ This surface modulation originates from the Gaussian energy distribution of the CO_2_ laser beam, the line-by-line scanning strategy, and rapid melt–quench dynamics during processing. Localized differences in energy density across each scan track lead to preferential sintering/melting at the beam center and differential shrinkage, resulting in a characteristic groove-like morphology. Similar features have also been observed in our previous work on nonreactive laser sintering of LLZTO. This laser-induced 3D surface topology may influence interfacial contact behavior in solid-state battery assemblies by modifying the effective contact area at solid–solid interfaces. In this context, increased surface roughness could be beneficial by enhancing interfacial contact and potentially reducing local current density. The 3D wavy surface morphology may enhance contact with electrode layers, potentially reducing effective current density and aligning with emerging concepts in three-dimensional solid-state battery architectures.^59,60^

Importantly, these surface features are not inherent limitations of the RLS process and can be tuned through laser parameters such as beam shaping, spot size, raster spacing, and scan speed, or further modified via postprocessing strategies including surface planarization or interfacial coating deposition. Overall, the observed morphology reflects a controllable outcome of the laser processing window, highlighting the distinct densification and surface evolution pathways enabled by reactive laser sintering compared with conventional furnace processing.

To further probe the microstructural differences induced by the sintering pathway, the samples were characterized by scanning electron microscopy (SEM), with emphasis on grain morphology and grain-boundary density. Figure 3 presents top-view and cross-sectional SEM images of furnace-sintered and RLS LLZTO (AED: 1.67 J mm^–2^). The furnace-sintered sample (Figure 3a) exhibits a relatively uniform fine-grained microstructure with closely packed grains and well-defined grain boundaries, characteristic of conventionally sintered LLZTO ceramics. In contrast, the RLS LLZTO (Figure 3b) displays significantly larger grains and a reduced grain-boundary density. Although the overall microstructure remains highly dense, the grain boundaries appear less distinct, and localized surface features or void-like regions are observed, likely arising from the rapid solidification and localized melt–quench dynamics associated with laser processing.

**Figure 3:**
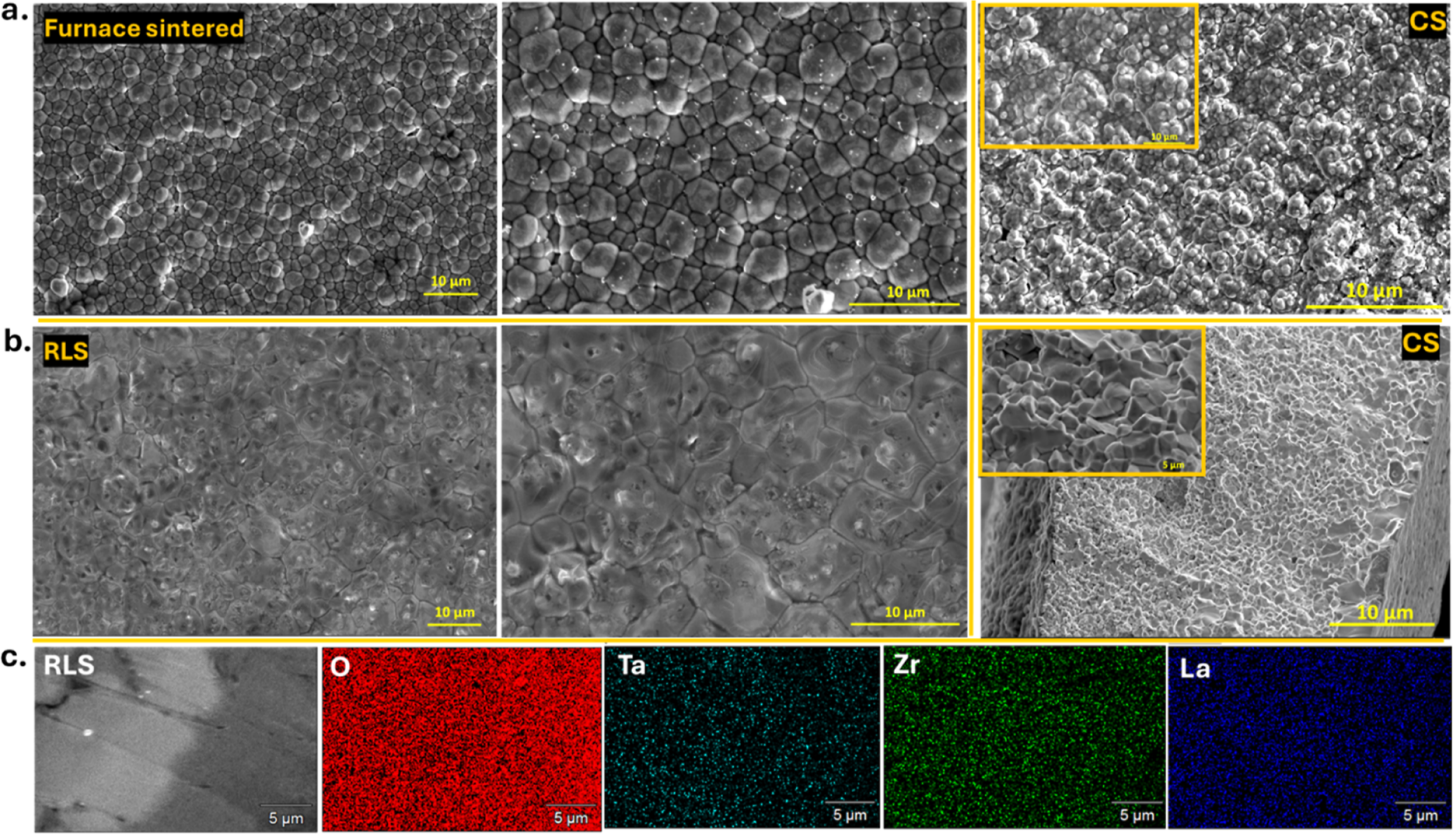
Microstructural characterization of LLZTO samples. SEM images of (a) furnace-sintered; (b) CO_2_ reactive laser sintering LLZTO using an areal laser energy density of 1.67 J mm^–2^, showing top-view and cross-sectional morphologies; and (c) EDS elemental maps of La, Zr, and Ta in an ion-polished RLS LLZTO cross-section.

These differences reflect the fundamentally distinct thermal conditions enabled by RLS, where extremely high heating and cooling rates generate steep thermal gradients and transient localized melting at particle interfaces. Consistent with previous reports on laser sintering of ceramics,^61,62^ such transient melt-assisted sintering enhances mass transport and grain-boundary mobility, promoting rapid pore elimination, grain coarsening, and densification within subsecond processing times. Simultaneously, rapid quenching suppresses prolonged diffusion and preserves nonequilibrium microstructural features formed during laser exposure.

Figure 3c shows EDS elemental mapping of an ion-polished RLS LLZTO cross-section, revealing an overall homogeneous distribution of La, Zr, and Ta throughout the microstructure. Quantitative EDS analysis (Figure S1) further indicates that the elemental composition is generally consistent with the nominal LLZTO garnet stoichiometry, with an approximate composition of Zr ≈ 1.54, La ≈ 2.70, Ta ≈ 0.76, and O ≈ 10.9 per formula unit.

Grain size analysis further highlights the influence of the sintering pathway on microstructural evolution. The furnace-sintered sample exhibits a smaller average grain size of 1.954 ± 0.556 μm (Figure 4a), whereas the RLS LLZTO displays a significantly larger average grain size of 5.833 ± 2.445 μm (Figure 4b), indicating enhanced grain growth under rapid laser sintering conditions. This pronounced coarsening in the RLS sample is attributed to the intense localized heating and steep thermal gradients generated during laser exposure, which accelerate atomic diffusion and grain-boundary mobility.^63–65^

**Figure 4:**
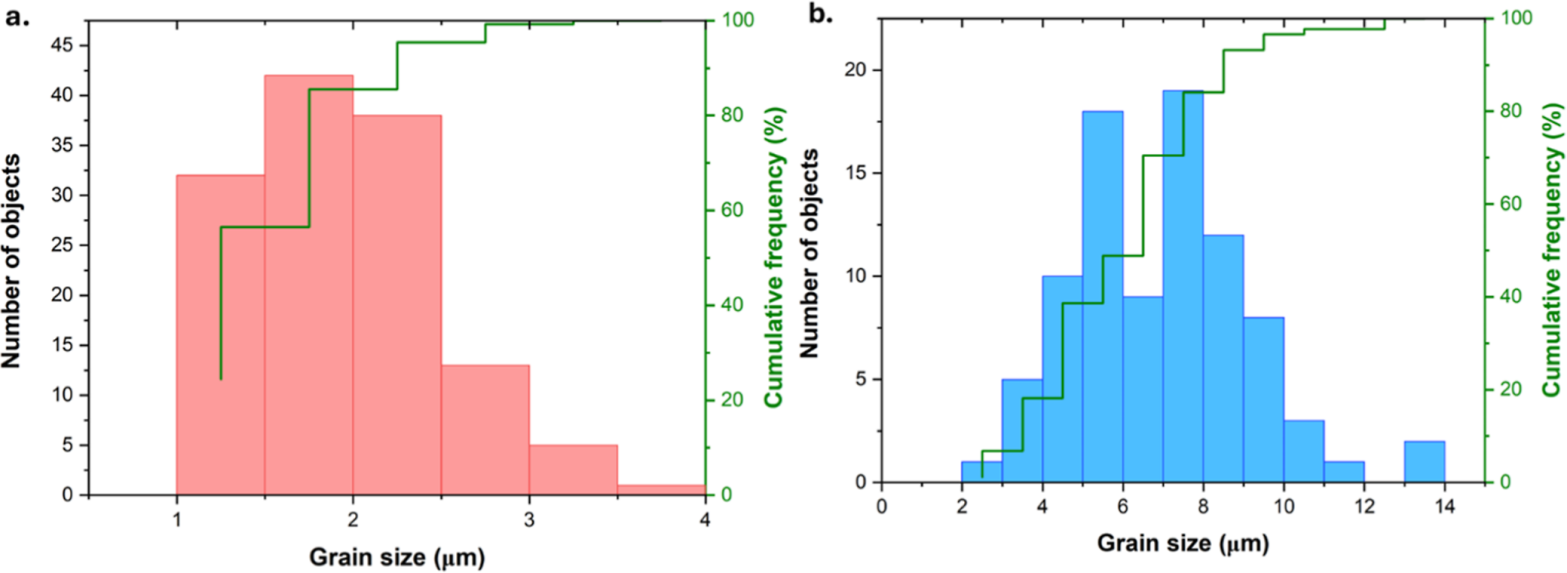
Histograms of (a) furnace-sintered LLZTO; and (b) reactive laser sintering LLZTO, showing the distribution of grain sizes.

The densification mechanism in RLS can be described as a hybrid process involving transient liquid-phase-assisted sintering and accelerated solid-state diffusion.^66,67^ Under intense CO_2_ laser irradiation, localized surface and interparticle melting occurs, forming short-lived liquid films at particle contacts. These transient liquid regions enhance capillary-driven mass transport, promoting rapid neck formation, pore elimination, and densification within subsecond time scales.^68^ Concurrently, steep thermal gradients and high peak temperatures promote accelerated solid-state diffusion in surrounding regions. The combination of these processes enables rapid grain growth and densification, while quenching freezes the evolved microstructure, preserving the resulted large-grained, highly dense morphology characteristic of RLS-processed LLZTO.

To further evaluate densification, representative samples from each processing route were ion-milled and analyzed by SEM cross sections using ImageJ to estimate porosity and relative density (Figure S2). The furnace-sintered LLZTO exhibits a porosity of 15.46 ± 1.96%, whereas the RLS sample shows a substantially lower porosity of 4.76 ± 0.27%, corresponding to a relative density of ∼95%. The comparatively lower density of the furnace-sintered sample relative to typical literature values (>90–95% at ∼1100 °C) may arise from the small pellet dimensions used in this study (<1 cm diameter and ∼500 μm thickness), which increase the surface-area-to-volume ratio and promote lithium volatilization during prolonged high-temperature sinterin.^69,70^ In contrast, the rapid RLS process enables efficient densification while minimizing lithium loss. It should be noted that porosity values derived from SEM cross sections may be influenced by localized sampling effects, particularly given the textured surface morphology of the RLS samples. To reduce this uncertainty, multiple representative cross-sectional regions were analyzed and averaged. Nevertheless, complementary bulk density measurements, such as Archimedes analysis, would provide additional validation and will be explored in future studies.

The mechanical properties of the RLS and furnace-sintered LLZTO samples were measured by nanoindentation (Figure 5). The RLS LLZTO exhibits a reduced modulus of 135.6 ± 6.2 GPa and a hardness of 8.08 ± 0.2 GPa at 1 μm contact depth, while the furnace-sintered sample shows comparable modulus of 134.8 ± 6.9 GPa and slightly higher hardness of 8.87 ± 0.2 GPa, respectively. The possible hardness reduction may arise from rapid sintering induced residual stress, which is described later in the X-ray diffraction (XRD) analysis. Overall, these results indicate that ultrafast reactive laser sintering preserves the mechanical integrity of the garnet framework while enabling rapid densification under localized thermal conditions.

**Figure 5:**
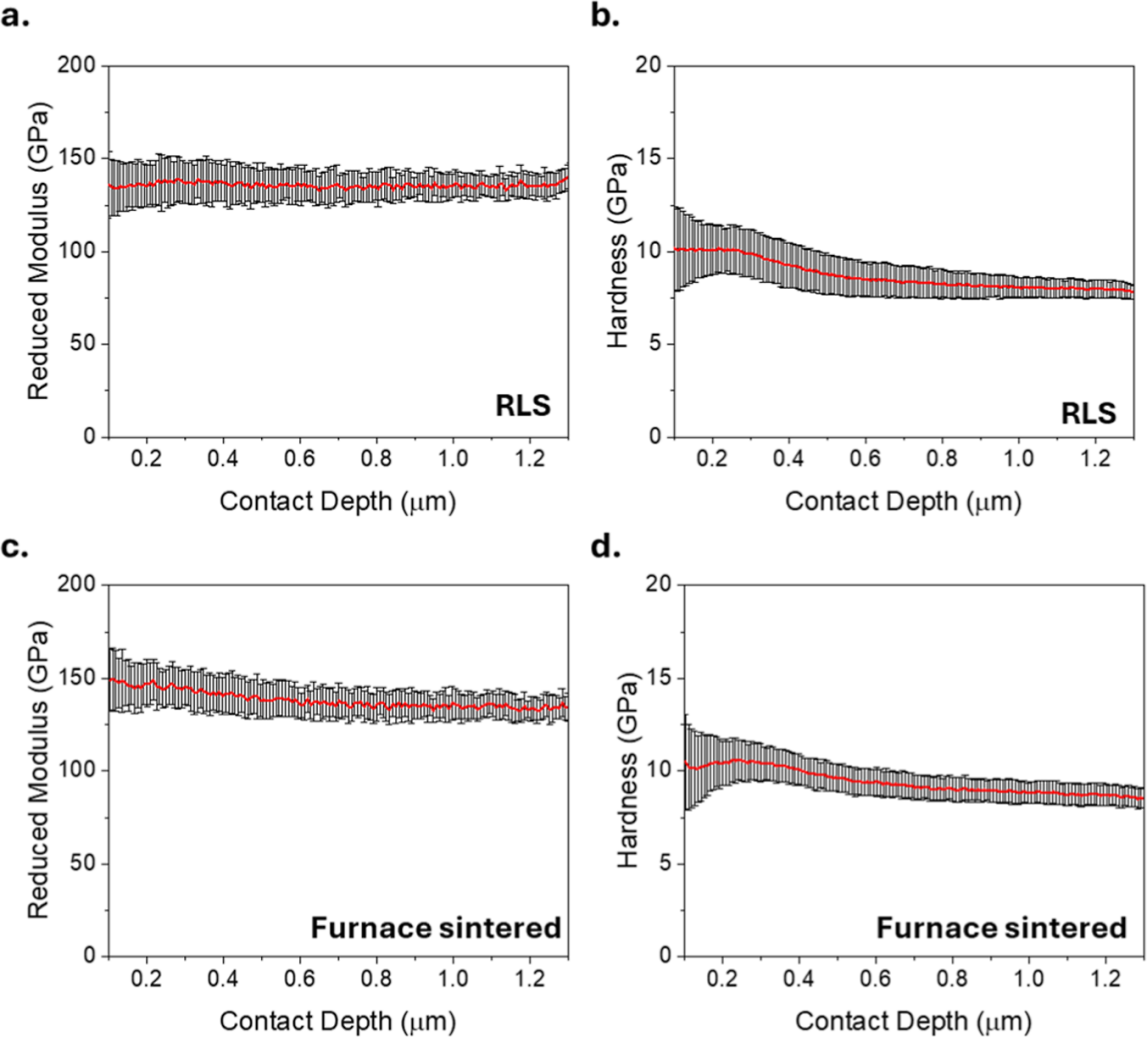
Nanoindentation-derived mechanical properties comparing the reduced modulus and hardness values of both samples of (a,b): reactive laser sintered LLZTO sample, and (c,d) furnace sintered LLZTO sample.

The furnace-sintered and RLS LLZTO samples were characterized by X-ray diffraction (XRD) to examine their phase composition and crystallographic structure. Figure 6a presents the XRD patterns of the ball-milled (BM) LLZTO precursors, the RLS-processed sample, and the furnace-sintered counterpart, with all Bragg reflections indexed to the cubic garnet phase (c-LLZTO). As expected, the high-energy wet-milled precursors exhibit residual unreacted phases, as shown in Figure S3. In contrast, both sintered samples display diffraction patterns consistent with the cubic LLZTO structure, confirming successful phase formation after sintering. Figure 6b shows the XRD pattern and Rietveld refinement of the RLS LLZTO processed at an areal energy density (AED) of 1.67 J mm^–2^, with the corresponding crystallographic parameters summarized in Table 1. The refinement confirms the formation of phase-pure cubic LLZTO, demonstrating the capability of reactive laser sintering to simultaneously induce phase formation and densification within ultrafast (<1 s) processing times. To investigate the influence of laser energy input on the LLZTO phase evolution and crystallographic ordering, the laser scan speed was decreased from 4 mm s^–1^ to 3 mm s^–1^, increasing the AED from 1.67 to 2.22 J mm^–2^. As shown in Figure 6c this increase in energy density produces noticeable changes in the diffraction pattern. In particular, the intensities of the (211) and (321) reflections decrease, the (022) reflection disappears, and the (004) reflection becomes more pronounced relative to both the lower-AED RLS sample and the furnace-sintered LLZTO (Figure 6b,d). These changes suggest that higher laser energy densities influence crystallographic texture and structural ordering during rapid laser processing.

**Figure 6:**
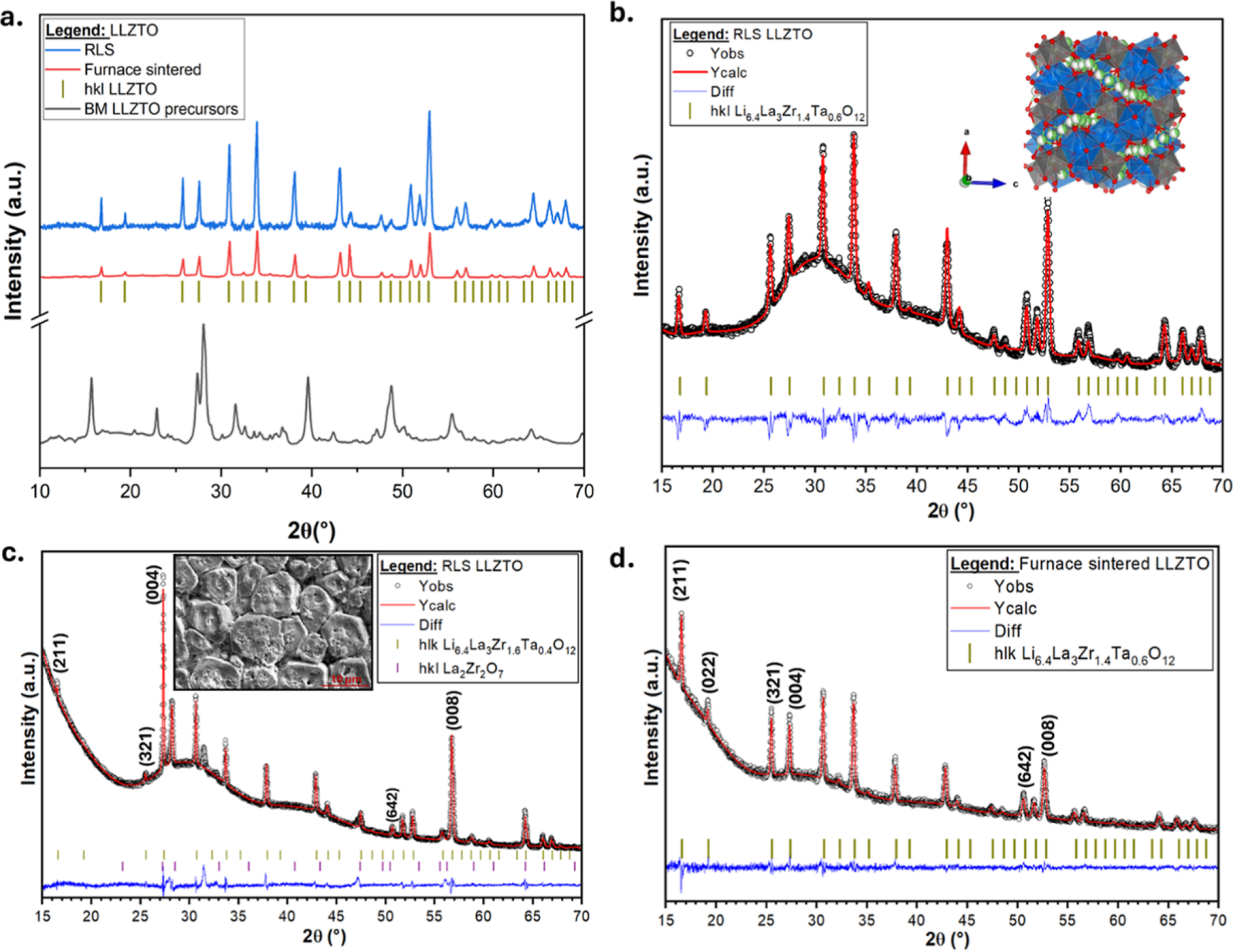
(a) X-ray diffraction patterns of ball-milled LLZTO precursors, furnace sintered LLZTO, and reactive laser sintering (RLS) LLZTO (AED: 1.67 J mm^–2^); (b) Rietveld refinement of the RLS LLZTO sample processed at an areal laser energy density of 1.67 J mm^–2^ yielded agreement factors (*R*_exp_ = 2.28, *R*_wp_ = 3.93, *R*_p_ = 2.88, GOF = 1.72). An inset representation of the refined cubic crystal structure is also shown. (c) Rietveld refinement of the RLS LLZTO sample processed at an areal laser energy density of 2.22 J mm^–2^ indicates the presence of 90.77 wt % cubic LLZTO and 9.23 wt % La_2_Zr_2_O_7_ (LZO), with refinement parameters of *R*_exp_ = 0.99, *R*_wp_ = 1.93, *R*_p_ = 1.14, and GOF = 1.95. An inset SEM image of the corresponding sample is also shown; (d) Rietveld refinement of the furnace sintered LLZTO *R*_exp_: 3.01, *R*_wp_: 2.28, *R*_p_: 2.10, GOF: 1.32. Black circles represent the experimental data points, the red line denotes the calculated pattern, and the difference curve is shown in blue.

**Table 1:** Atomic Coordinates, Site Occupation Factor, and Isotropic Displacement Parameters of Reactive Laser Sintering Li_6.4_La_3_Zr_1.4_Ta_0.6_O_12_ ^a^

chemical formula	Li_6.46_La_3_Zr_1.466(116)_Ta_0.522(62)_O_12_
crystal system	cubic
space group	*Ia*3̅*d* (no. 230)
lattice parameters	*a* = *b* = *c* = 12.9363(59) Å, *V* = 2164.88 (30) Å^3^, *Z* = 8

atom	Wyckoff site	*x*	*y*	*Z*	SOF	*U* (eq) (Å^2^)
O1	*96h*	0.09198(92)	0.1877(10)	0.28902(79)	1	**0.00899**
Ta1	*16a*	0	0	0	0.261(31)	**0.00470**
Zr1	*16a*	0	0	0	0.733(58)	**0.00470**
La1	*24c*	1/4	1/8	0	1	**0.00650**
Li1	*96h*	0.0892(70)	0.2049(76)	0.3873(83)	0.4	**0.01900**
Li2	*24d*	1/4	3/8	0	0.56	**0.01200**

a After the scale factor, Chebyshev background, peak shape, lattice parameters, and atomic positions were refined. The SOFs and atomic displacements parameters were fixed.

At the higher AED (2.22 J mm^–2^), a La_2_Zr_2_O_7_ secondary phase (∼9.23 wt %) is also detected, likely due to partial garnet decomposition under highly localized and nonequilibrium thermal conditions. Complementary SEM analysis (inset Figures 6c and S4) reveals a densely sintered microstructure consisting of coalesced faceted grains with localized surface roughening and edge rounding, further supporting enhanced localized melting at elevated laser energy density. Together, these results demonstrate that laser processing parameters strongly influence phase stability, microstructure, and crystallographic texture, highlighting the tunability of the RLS approach.

The Williamson–Hall (W–H) method using the Uniform Deformation Model (UDM)^71^ was employed to estimate the crystallite size and lattice microstrain of the RLS and furnace-sintered LLZTO samples, with the corresponding plots shown in Figure 7a,b. In this approach, peak broadening is assumed to originate from the combined contributions of finite crystallite size and lattice strain, following the relation

$$\beta \cos\theta =\frac{k\lambda }{D}+4\varepsilon \sin\theta$$

where β is the full width at half-maximum (fwhm) after instrumental broadening correction, θ is the Bragg angle, *D* is the crystallite size, and ε is the lattice microstrain. The analysis was performed assuming isotropic lattice deformation and Lorentzian peak broadening, consistent with previous reports employing the UDM Williamson–Hall approach for defect and strain analysis in oxide nanomaterials. Although the method provides an approximate estimation of crystallite size and microstrain, it remains useful for comparative analysis between samples processed under identical experimental conditions.

**Figure 7:**
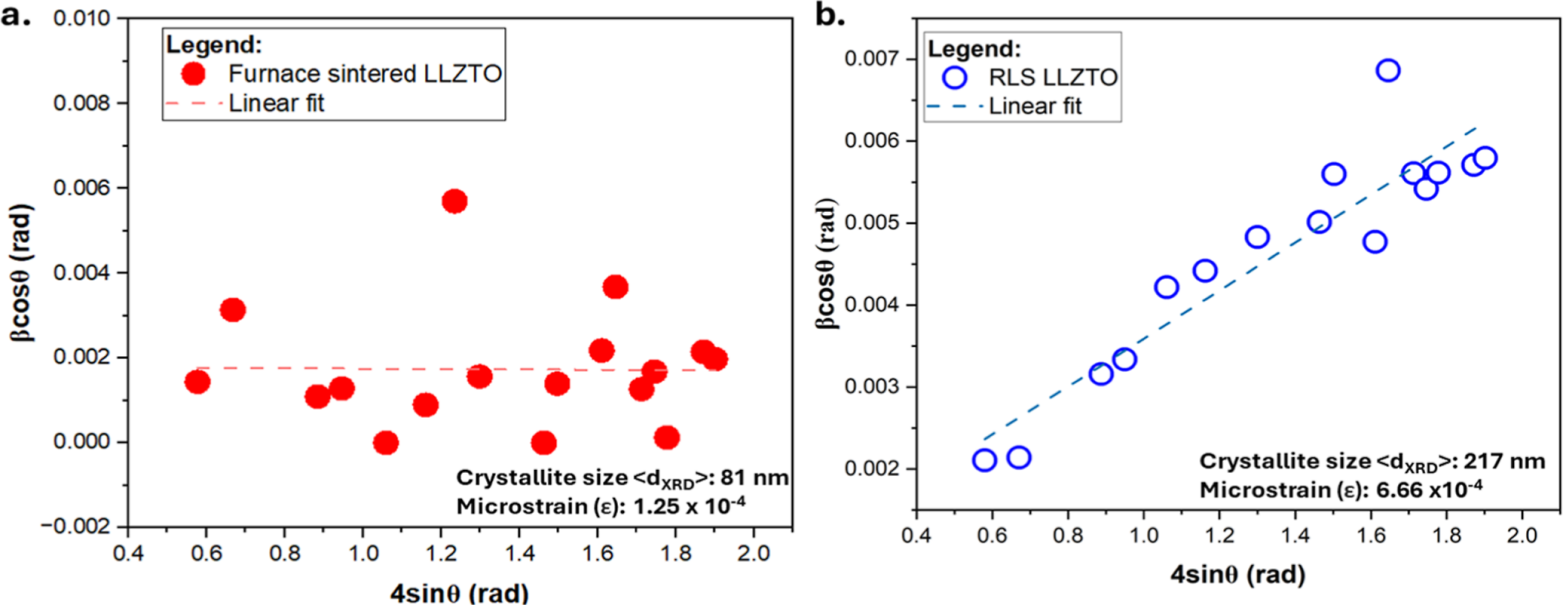
Williamson–Hall plots in the uniform deformation model of LLZTO samples. (a) furnace-sintered; and (b) reactive laser sintering LLZTO.

Compared to the furnace-sintered sample, RLS LLZTO exhibits a significantly larger crystallite size (217 nm vs 82 nm), indicative of enhanced grain coarsening under ultrafast laser processing conditions. However, the RLS sample also exhibits a higher lattice microstrain than the furnace-sintered counterpart, as determined from the slope of the Williamson–Hall plots (Figure 7) (6.66 × 10^–4^ for RL LLZTO vs, 1.25 × 10^–4^ for furnace-sintered LLZTO). The increased microstrain in the RLS sample likely originates from the extreme thermal gradients and rapid melt–quench dynamics associated with localized laser heating, which can introduce residual lattice distortions and nonequilibrium structural defects.

Figure 8a shows the Raman spectra of furnace-sintered and RLS LLZTO samples. All peaks of both samples can be indexed to the cubic LLZTO phase (c-LLZTO). Raman was collected on both sides of the RLS LLZTO pellet (front and back) to verify film homogeneity. In the spectra, a narrow band in the low-frequency region (<200 cm^–1^) corresponds to La cation vibrations. Vibrational modes below 300 cm^–1^ are associated with the LiO_6_ octahedral units (96h Li1 position), while bending modes between 300 and 550 cm^–1^ are assigned to the LiO_4_ tetrahedral units (24d Li2 position). The peaks at ∼648 and ∼736 cm^–1^ are attributed to the stretching modes of ZrO_6_ and TaO_6_ octahedra (16a positions), consistent with previous reports. In contrast to XRD, Raman spectroscopy detects a small signal at 1100 cm^–1^ in the RLRS LLZTO sample, assignable to Li_2_CO_3_ present at very low concentration -below the detection limit of XRD. Given the surface sensitivity of Raman, this signal likely arises from Li_2_CO_3_ located at the pellet surface, either due to brief exposure to air during handling or storage. The presumably low concentration of Li_2_CO_3_ in the RLS LLZTO sample is not expected to significantly impact its ionic conductivity, as discussed below.

**Figure 8:**
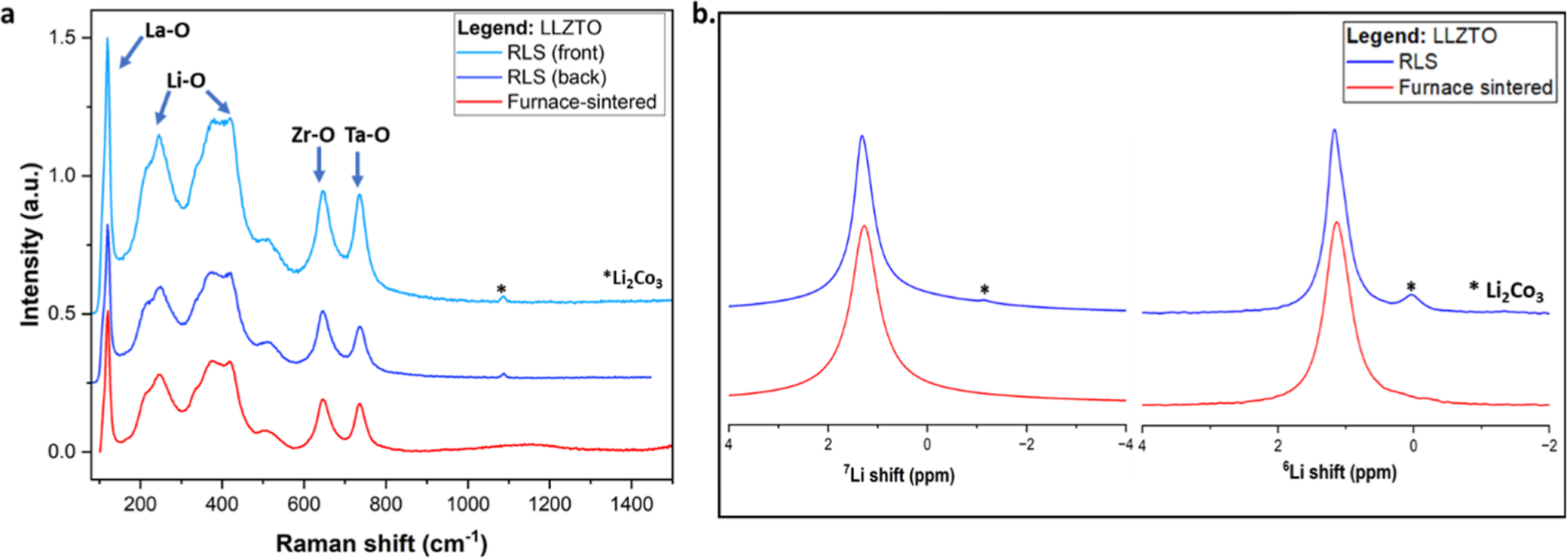
Characterization of furnace-sintered and reactive laser sintering LLZTO. (a) Raman spectra of both samples, collected on the front and back of the RLS pellet; and (b) solid-state ^6^Li and ^7^Li NMR spectra of both samples.

Complementary ^6^Li and ^7^Li solid-state nuclear magnetic resonance (NMR) spectra (Figure 8b) confirm the formation of c-LLZTO, with dominant peaks corresponding to the cubic phase. In addition, a minor upfield peak is observed, consistent with a nonionically conductive Li species, likely Li_2_CO_3_. Although NMR is generally a bulk-sensitive technique, small amounts of surface Li_2_CO_3_ adsorbed on grain boundaries can also produce detectable signals, explaining the correlation with Raman. While CO_2_ laser processing has been shown to decompose surface Li_2_CO_3_,^58^ this aspect is beyond the scope of the present study.

The ion transport behavior of both RLS and furnace-sintered LLZTO samples was investigated by AC impedance spectroscopy. Figure 9a,b present the Nyquist plots collected at 30 °C for the furnace-sintered and RLS LLZTO samples, respectively. Both spectra consist of a high-frequency semicircle followed by a low-frequency Warburg tail, characteristic of predominantly ionic conduction. The ionic conductivity of both samples follows Arrhenius behavior over the investigated temperature range, and the corresponding activation energies for Li^+^ diffusion were extracted from the linear fits shown in Figure 9c. Full temperature-dependent impedance spectra (30–80 °C) are provided in the insets of Figure 9a,b.

**Figure 9:**
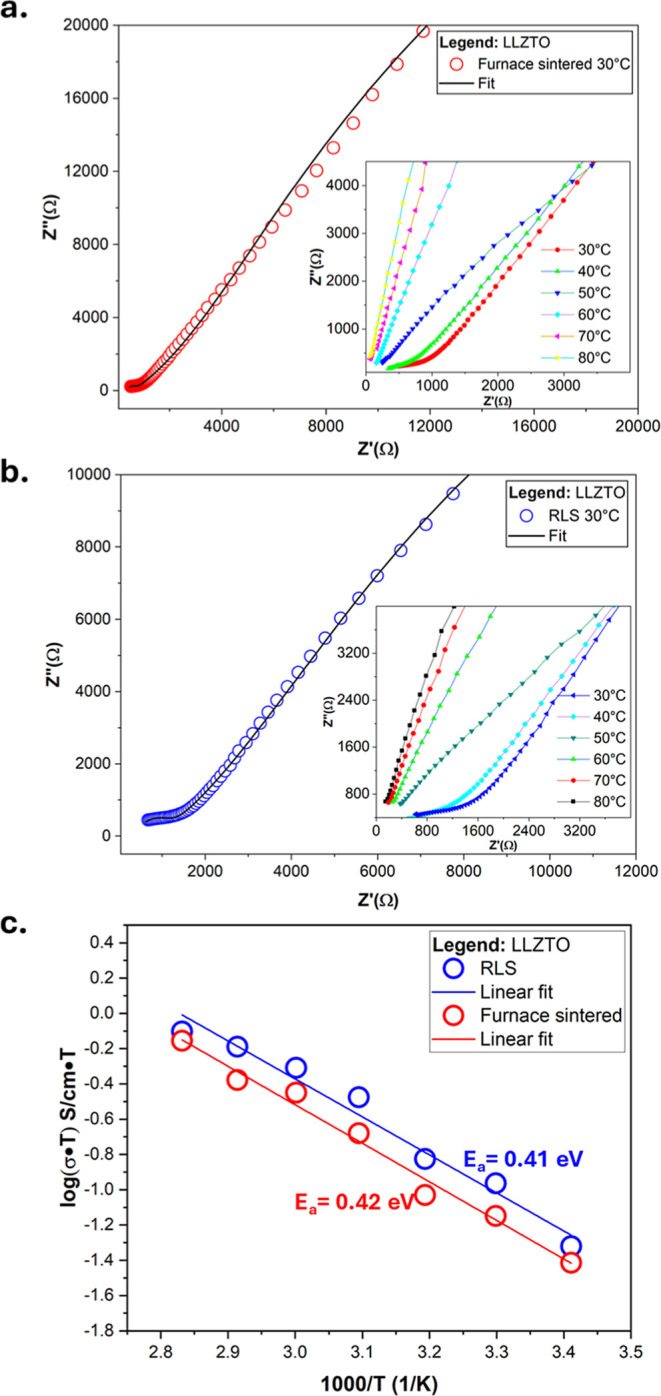
AC impedance analysis of LLZTO samples. Nyquist plots of (a) furnace-sintered; (b) reactive laser sintering LLZTO; insets show full temperature-dependent Nyquist spectra (30–80 °C); and (c) Arrhenius plots of ionic conductivity and extracted activation energies.

The RLS LLZTO exhibits a slightly higher ionic conductivity than the furnace-sintered sample, reaching 0.36 ± 0.08 mS cm^–1^ compared to 0.23 ± 0.12 mS cm^–1^, while both samples display similar activation energies (0.41 eV for RLS LLZTO and 0.42 eV for furnace-sintered LLZTO). These conductivity values are comparable to those reported for Ta-doped LLZTO films fabricated via ultrafast Joule-heated carbon strip sintering, which typically exhibit ionic conductivities in the range of 0.1–1 mS cm^–1^.^72^ Although separate bulk and grain-boundary resistances could not be fully deconvoluted due to overlap of the impedance semicircles within the measured temperature range, the improved conductivity of the RLS sample is consistent with its higher relative density and larger crystallite size, both of which reduce grain-boundary resistance and facilitate Li^+^ transport. Notably, despite exhibiting slightly higher lattice microstrain than the furnace-sintered sample, the RLS LLZTO still demonstrates superior ionic conductivity, indicating that the beneficial effects of enhanced densification and crystallite coarsening outweigh the moderate increase in lattice distortion. Collectively, these results demonstrate that reactive laser sintering enables favorable microstructural evolution for Li^+^ transport while minimizing prolonged thermal exposure and lithium volatilization, highlighting RLS as a promising ultrafast processing strategy for high-performance garnet solid electrolytes.

## 3 Conclusion

In this work, we demonstrate that reactive laser sintering (RLS) provides a rapid and scalable route for fabricating LLZTO solid electrolytes through simultaneous phase formation and densification. Compared with conventional furnace sintering, RLS enables ultrafast densification on subsecond time scales, achieving ∼95% relative density while minimizing lithium loss and limiting secondary phase formation. Comprehensive characterization by SEM, EDS, X-ray diffraction (XRD), Raman spectroscopy, and solid-state ^6^Li/^7^Li NMR confirms the formation of cubic garnet LLZTO with homogeneous microscale elemental distribution and dense microstructures.

Microstructural analysis reveals that RLS promotes significantly larger grain and crystallite sizes relative to furnace-sintered LLZTO, consistent with enhanced densification and reduced grain-boundary density. Although the RLS samples exhibit slightly higher lattice microstrain, they demonstrate improved ionic conductivity (0.36 mS cm^–1^) compared to conventionally sintered samples (0.23 mS cm^–1^), indicating that the beneficial effects of densification and grain coarsening outweigh the moderate increase in lattice distortion. In addition, nanoindentation measurements show that RLS preserves the mechanical integrity of the garnet framework despite the highly localized ultrafast thermal processing conditions.

Overall, reactive laser sintering integrates phase formation, densification, and microstructural control into a single ultrafast processing step, establishing a promising manufacturing strategy for garnet-type solid electrolytes. Beyond improving LLZTO processing efficiency and electrochemical performance, this approach provides a pathway toward scalable and high-throughput fabrication of oxide solid electrolytes for next-generation solid-state batteries.

## 4 Experimental Section

### 4.1 Synthesis

LiOH·H_2_O (battery grade, Sigma-Aldrich), La_2_O_3_ (99.99%, Sigma-Aldrich), ZrO_2_ (99.7%, Sigma-Aldrich), Ta_2_O_5_ (Sigma-Aldrich) and 5 wt % excess of LiOH·H_2_O -for compensating lithium loss during sintering-were weighed in stoichiometric ratios and ball milled using a using a zirconia milling jar (SPEX SamplePrep) in isopropanol at 1700 rpm for 1 h with 3 mm yttria-stabilized zirconia (YSZ) beads (1:14 ball to powder ratio). The powder was pressed at 15 MPa for 2–3 min to form pellets for RLS. The RLS LLZTO was annealed using a CO_2_ laser with wavelength λ = 10.6 μm, maximum power of 20 W and a Gaussian focal diameter of 1.5 mm. Samples were placed on a graphite foil in a chamber with continuous Argon gas purging and a resistive heating stage (1″ in diameter effective heating area). Stage heating was controlled by increasing the current through a Labview program. The ramping rate was approximately 30 °C/min. Once the stage temperature reached 1000 °C, laser was turned on and scanned across the sample with speed controlled by scanning galvo mirror system. After laser treatment, the stage was cooled down to room temperature and the sample was removed from the chamber. The reactive laser synthesis of c-LLZTO was performed using a 2 W CO_2_ laser, a 1.5 mm (e^–2^) Gaussian beam diameter, a scan speed of 4 mm s^–1^, and a hatch spacing of 0.3 mm, corresponding to an aerial laser energy density of 1.67 J mm^–2^. For samples processed at an energy density of 2.22 J mm^–2^, a 2 W CO_2_ laser was used with a Gaussian beam (1.5 mm e^–2^ diameter), a scan speed of 3 mm s^–1^, and a hatch spacing of 0.3 mm. For the LLZTO samples prepared by conventional furnace methods, the pellets were sintered at 1100 °C for 6 h at a heating rate of 5 °C·min^–1^.

### 4.2 Nanoindentation Measurements

iO4 Femto-indenter equipped with Diamond Berkovich tip was used to conduct nanoindentation experiments on reactive laser sintered and furnace sintered LLZTO samples. Both samples were mechanically polished with sandpapers up to grid #1200 in the final step. Both polishing and indentation were carried out inside an Ar-filled glovebox to avoid Li_2_CO_3_ surface layer formation due to air exposure. Reduced modulus and hardness values were reported for 0.9–1.1 μm contact depth range, averaging 25 data sets.

### 4.3 X-ray Diffraction and Raman Characterization

X-ray diffraction of the reactive laser sintered and furnace sintered LLZTO samples was performed using a Bruker D8 Advanced instrument operated at 40 kV and 40 mA with Cu–Kα radiation (λ = 1.54 Å) for phase identification, Rietveld quality pattern was recorded in Bragg–Brentano geometry. The samples were prepared in a glovebox using airtight sample holders (Bruker) to maintain an argon environment during testing. Rietveld refinements of the LLZTO samples were performed using the models for the LLZTO SSE reported by Kataoka and Akimoto.^73^ Rietveld refinements of the crystal structure including scale factor, Chebyshev background, peak shape, and lattice parameters, were simultaneously refined using the software package TOPAS 6­(Bruker-AXS). Refinement constraints that were used were as follows: the atomic coordinates and atomic displacement parameters were fixed to be the same of those obtained from reported model. Raman spectra were collected using an inVia confocal Raman microscope (Renishaw) equipped with a 633 nm He–Ne laser for excitation. The laser was focused onto the sample surface through a 50× objective lens. Spectra were acquired over the range of 100–1400 cm^–1^ with an acquisition time of 10 s per scan, and multiple accumulations were averaged to improve signal-to-noise ratio. The spectrometer was calibrated using the Si reference peak at 520.7 cm^–1^ prior to measurements. Data processing, including baseline correction and peak fitting, was performed using Renishaw WiRE software. Crystallite size and lattice microstrain were estimated using the Williamson–Hall (W–H) method within the Uniform Deformation Model (UDM). Unlike the Debye–Scherrer approach, the W–H method accounts for peak broadening contributions from both finite crystallite size and lattice strain. In the UDM approach, plots of β cos θ versus 4 sin θ were linearly fitted, where the slope corresponds to the lattice microstrain (ε) and the *y*-intercept was used to calculate the crystallite size (*D*) of the RLS LLZTO and furnaced sintered LLZTO samples.

### 4.4 Electrochemical Impedance Spectroscopy

The reactive laser sintered and furnace sintered LLZTO samples were first treated according to the method reported by Ruan et al.^74^ Each LLZTO sample was first immersed in a solution of phosphoric acid and ethanol (1:1 vol ratio) for 3 min to convert any Li_2_CO_3_ on the surface into Li_3_PO_4_. The sample was then washed with ethanol twice for 2 min each and dried under vacuum for 20 min before assembling into a cell in an argon-filled glovebox. Each side of the LLZTO pellets was coated with silver paint to establish electronic contact. No external stack pressure was applied during conductivity measurements for either the laser-sintered or furnace-sintered samples. AC impedance spectroscopy was carried out using a VMP3 potentiostat/galvanostat (Bio-Logic Science Instruments) over a frequency range of 1 Hz to 1 MHz. Ionic conductivity (σ) was calculated using σ = *t*/(*RA*), where *R* is the bulk resistance extracted from the high-frequency intercept, *t* is the pellet thickness, and *A* is the electrode area. The impedance spectra were fitted using the equivalent circuit R1 + C1/R2 + C2/R3 + Wd, where R1 represents the bulk resistance of the pellet, C1 and R2 account for the grain-boundary capacitance and resistance, C2 and R3 model interfacial or surface contributions, and Wd3 corresponds to a Warburg element representing ion diffusion within the material. The use of silver paste to establish electronic contact may contribute to the interfacial response captured by the circuit. Activation energies were determined from Arrhenius analysis of the temperature-dependent conductivity.

### 4.5 Scanning Electron Microscopy

The microstructure of the furnace-sintered and laser-sintered LLZTO samples was characterized using a scanning electron microscope (SEM). Samples were mounted on aluminum stubs using conductive carbon tape. Top-view and cross-sectional images were acquired to evaluate grain morphology, grain boundaries, and porosity. Grain size analysis was performed using ImageJ software. Individual grains were manually or semiautomatically outlined, and the area of each grain was measured to generate histograms of grain size distributions. Cross-sectional samples of furnace-sintered and laser-sintered (RLS) LLZTO pellets were prepared by ion milling to expose smooth surfaces for SEM imaging. A few representative samples were analyzed for each sintering approach, and multiple regions were imaged per sample to ensure statistical relevance. Porosity was estimated from the SEM images by outlining visible pores in ImageJ, measuring their areas, and calculating the ratio of total pore area to the total analyzed area. The relative density of each sample was then calculated as 100% minus the porosity, and the average values were determined for each sintering method.

### 4.6 Ar Ion Milling

Samples were prepared for SEM imaging using a JEOL Ar ion mill. The samples were fractured to expose a clean surface and then mounted onto a Si chip with carbon tape. An accelerating voltage of 6 kV was used with an ion current between 100 and 150 μA. Milling was alternated on/off for 10 s/10 s, respectively, using a total milling time of 1 h to lower the sample temperature during milling. The ion milling resulted in a milled area with a width approximately 400 μm across the thickness of the sample.

### 4.7 Solid-State NMR Spectroscopy

Solid-state ^6^Li and ^7^Li NMR spectroscopy was performed on a Bruker Avance III 400 MHz NMR spectrometer equipped with a 4 mm double-resonance magic-angle spinning (MAS) probe. Samples were packed into 4 mm zirconia rotors and spun at a MAS frequency of 12 kHz to minimize anisotropic broadening. Single-pulse experiments were conducted using a π/2 pulse length of 2.5 μs (^7^Li) and 3.0 μs (^6^Li), a recycle delay of 2 s, and 256–1024 scans depending on signal intensity. Chemical shifts were referenced to 1 M LiCl aqueous solution at 0 ppm. All spectra were processed using Bruker TopSpin software, with baseline correction and phase adjustment applied.

## Supplementary Material



## Notes

EDS elemental analysis of RLS LLZTO (SEM regions, spectra, and quantitative composition tables); cross-sectional SEM images of ion-milled furnace-sintered and RLS LLZTO pellets; X-ray diffraction patterns of ball-milled precursors and simulated reference phases (PDF)
