# Supplementary material for: Ultrafast Reactive Laser Sintering of Highly Conductive Garnet-Type LLZTO Solid Electrolytes
Source: ACS Appl Mater Interfaces. 2026 Jul 6;18(27):37654–65. doi: 10.1021/acsami.6c05262 (PMC13383265; doi:10.1021/acsami.6c05262)

Supporting Information

Ultrafast Reactive Laser Sintering of Highly Conductive Garnet-Type LLZTO Solid Electrolytes

Erika P. Ramos,[1,*] *Yudan Chen,*[2] *Aaron Santomauro,*[3] *Yan-Yan Hu,*[2] *Samuel Sanghyun Lee,*[4] *Jianhua Tong,*[3] *Jianchao Ye*[1,*]

[1]Materials Science Division, Lawrence Livermore national Laboratory, Livermore, CA 94550, USA

[2]Department of Chemistry & Biochemistry, Florida State University, Tallahassee, FL 32306, USA

[3]Department of Materials Science and Engineering, Clemson University, Clemson, SC 29634, USA

[4]Mechanical Engineering, Stanford University, Stanford, California 94305, USA

Co-responding authors

Erika P. Ramos, Materials Science Division, Lawrence Livermore National Laboratory; ramosguzman1@llnl.gov

Jianchao Ye, Materials Science Division, Lawrence Livermore National Laboratory; ye3@llnl.gov

Figure S1. **EDS analysis of RLS LLZTO: SEM image indicating the two analyzed areas, corresponding EDS spectra (keV vs. counts), and tables summarizing the elemental composition in weight % and atomic %.**

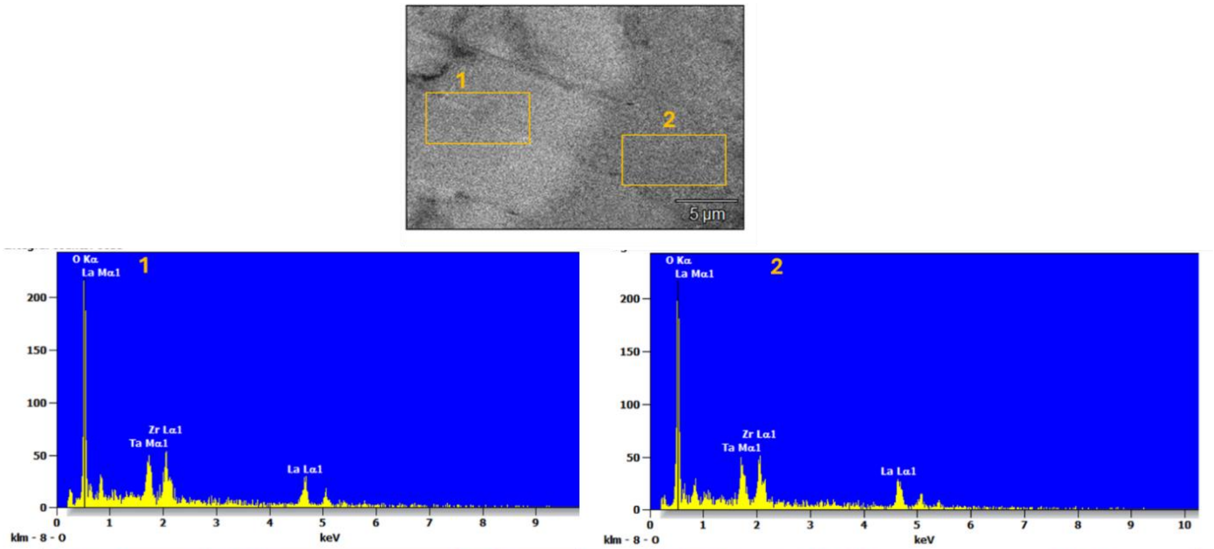

Element	Weight %	Atom %
O	22.1	69.6
Zr	19.1	10.6
La	40.8	14.8
Ta	18.0	5.0
Total	100.0	100.0

Element	Weight %	Atom %
O	20.1	67.5
Zr	14.8	8.7
La	49.9	19.3
Ta	15.2	4.5
Total	100.0	100.0

Figure S2. Cross-sectional SEM images of Ar ion-milled LLZTO pellets: a. furnace-sintered and b. reactive laser-sintered (RLS).

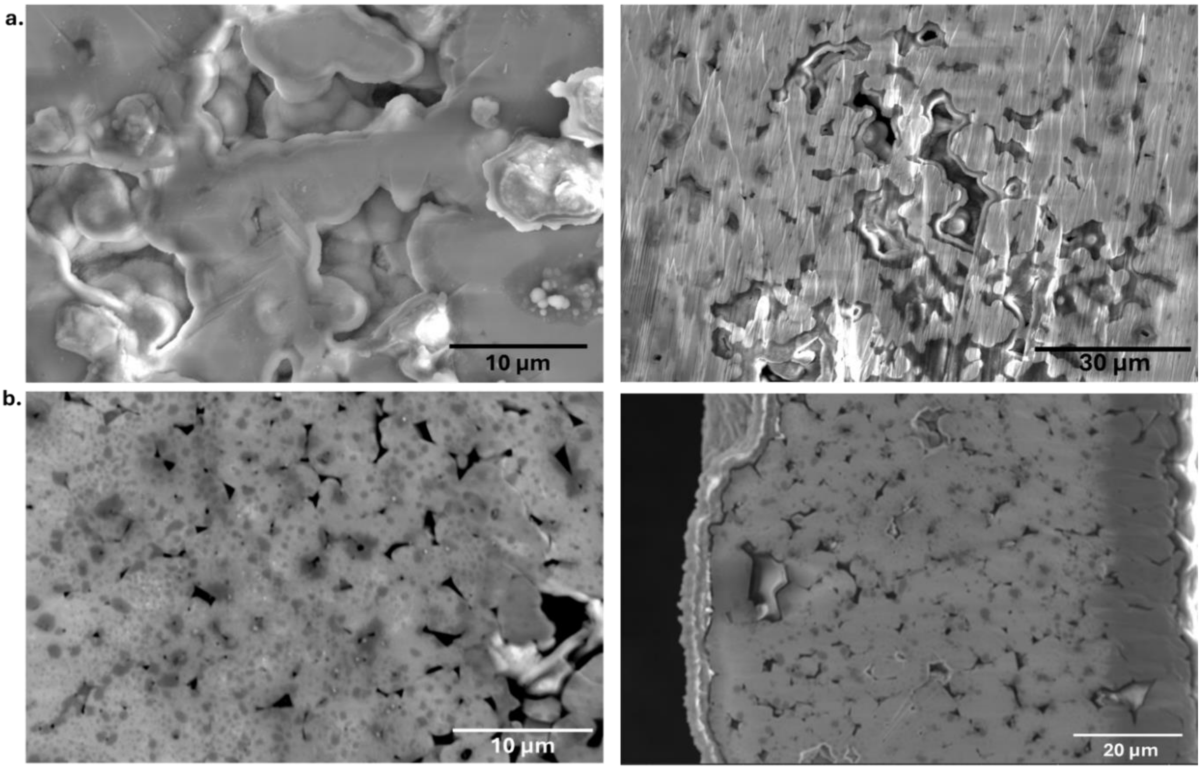

Figure S3. X-ray diffraction patterns of the ball-milled (BM) LLZTO precursors, and the simulated patterns of the employed precursors.

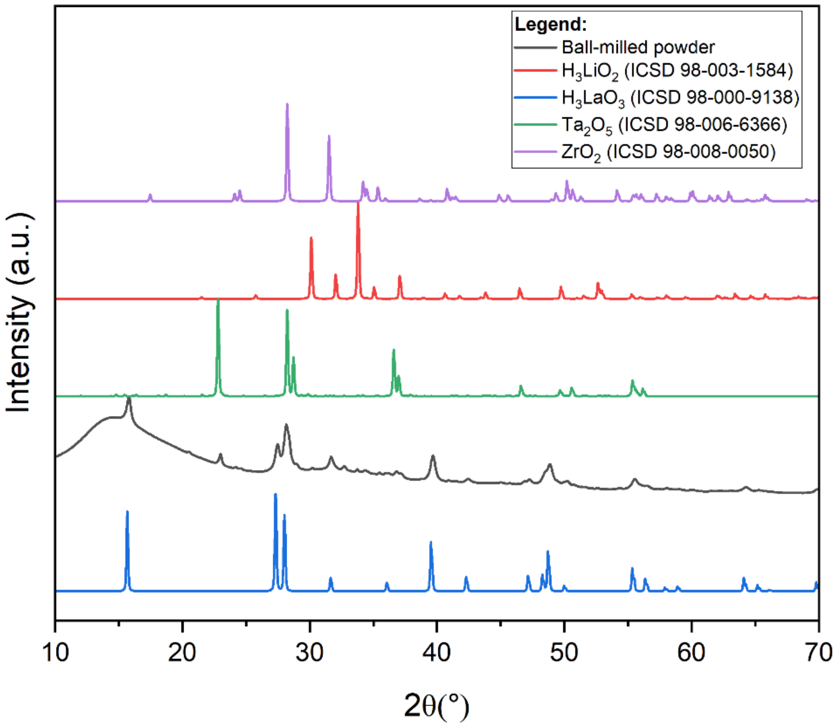

Figure S4. Top view and cross-section (CS) SEM images of reactive lase sintering of LLZTO processed at an areal laser energy density of 2.22 J mm^{-2}

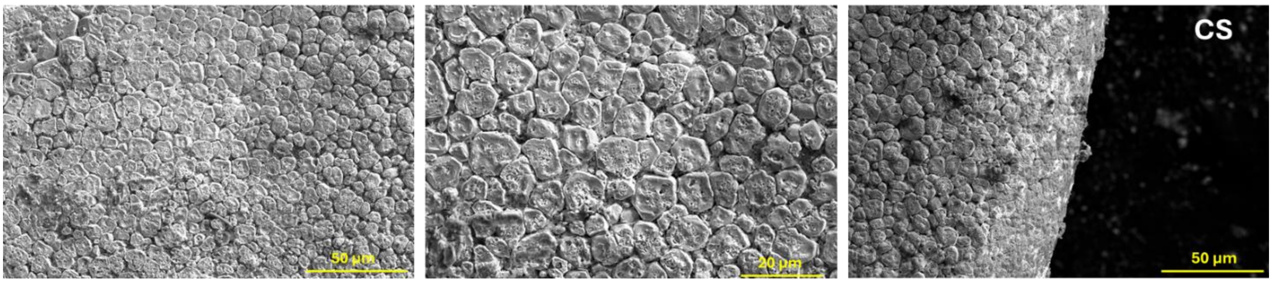

Supplement: Supplementary file 1 [file am6c05262_si_001.pdf]
